# Crystal Structure of the Shrimp Proliferating Cell Nuclear Antigen: Structural Complementarity with WSSV DNA Polymerase PIP-Box

**DOI:** 10.1371/journal.pone.0094369

**Published:** 2014-04-11

**Authors:** Jesus S. Carrasco-Miranda, Alonso A. Lopez-Zavala, Aldo A. Arvizu-Flores, Karina D. Garcia-Orozco, Vivian Stojanoff, Enrique Rudiño-Piñera, Luis G. Brieba, Rogerio R. Sotelo-Mundo

**Affiliations:** 1 Centro de Investigación en Alimentación y Desarrollo, A.C. Hermosillo, Sonora, México; 2 Departamento de Ciencias Químico Biológicas, Universidad de Sonora, Hermosillo, Sonora, México; 3 National Synchrotron Light Source, Brookhaven National Laboratory, Upton, New York, United States of America; 4 Departamento de Medicina Molecular y Bioprocesos, Instituto de Biotecnología-Universidad Nacional Autónoma de México, Cuernavaca, Morelos, México; 5 Laboratorio Nacional de Genómica para la Biodiversidad, Centro de Investigación y Estudios Avanzados, Irapuato, Guanajuato, México; The University of North Carolina at Chapel Hill, United States of America

## Abstract

DNA replication requires processivity factors that allow replicative DNA polymerases to extend long stretches of DNA. Some DNA viruses encode their own replicative DNA polymerase, such as the white spot syndrome virus (WSSV) that infects decapod crustaceans but still require host replication accessory factors. We have determined by X-ray diffraction the three-dimensional structure of the Pacific white leg shrimp *Litopenaeus vannamei* Proliferating Cell Nuclear Antigen (*Lv*PCNA). This protein is a member of the sliding clamp family of proteins, that binds DNA replication and DNA repair proteins through a motif called PIP-box (**P**CNA-**I**nteracting **P**rotein). The crystal structure of *Lv*PCNA was refined to a resolution of 3 Å, and allowed us to determine the trimeric protein assembly and details of the interactions between PCNA and the DNA. To address the possible interaction between *Lv*PCNA and the viral DNA polymerase, we docked a theoretical model of a PIP-box peptide from the WSSV DNA polymerase within *Lv*PCNA crystal structure. The theoretical model depicts a feasible model of interaction between both proteins. The crystal structure of shrimp PCNA allows us to further understand the mechanisms of DNA replication processivity factors in non-model systems.

## Introduction

Proliferating Cell Nuclear Antigen (PCNA) is a member of the sliding clamp family of DNA-replication accessory proteins. Their functions are critical to processes such as cell cycle control, chromatin remodeling, gene expression, apoptosis, and DNA repair [1], [2], [3], [4]. In most organisms PCNA is a homotrimer, in which its three subunits adopt a doughnut-shaped structure in a head-to-tail arrangement; this toroidal structure is extremely conserved in protozoa, humans, yeast and plants [5], [6], [7], [8], [9]. In bacteria, the PCNA homologue is called β clamp, that is formed by a homodimeric assembly with a six-fold symmetry forming a toroidal structure similar to most PCNAs reported [10]. Only few organisms have a non-canonical homotrimeric structure as in the crenarchaeon *Sulfolobus solfataricus* and in the model plant *Arabidopsis*, where their PCNA are formed by heterotrimers [11], [12].

The structure of PCNA is comprised by two α+β domains joined by an inter-domain connecting loop (IDCL) [7]. The PCNA molecule interacts with DNA by the inner face of the ring, which is composed by α-helices. Therefore, the arrangement of the α-helices in each monomer leads to a pseudo six-fold symmetry in the trimer comprised of 12 α-helices [13]. The inner face of the toroid has an array of basic residues positioned to provide favorable electrostatic interactions with the DNA-phosphate backbone. This structure allows PCNA to slide freely on DNA, once is assembled into DNA by the clamp loading complex [14].

In most cases, PCNA-interacting proteins contain a short sequence motif called PIP-box, which makes hydrophobic contacts with PCNA and has a consensus amino acid sequence QXX(M/L/I)XX(F/Y)(F/Y) [15]. However, there is also a novel PCNA-interacting motif (APIM) with an apparent consensus amino acid sequence MD(L/R)W(L/V/I)2(K/R) which is present in proteins involved in DNA repair and cell cycle control during genotoxic stress, the APIM motif was identified by bioinformatics analysis in about 200 nuclear proteins [16]. PCNA interacts with multiple protein partners and despite each PCNA binding protein has its specific contact site, most of them bind mainly through hydrophobic pocket formed by the IDCL, central loop and C-terminus in PCNA [17].

It is known that some viruses encode their own DNA polymerases and processivity factors as observed in T4 and RB69 bacteriophages or human viruses like herpes simplex and cytomegalovirus [18]. However, in some cases, pathogens like the Simian Virus 40 and bacteriophage T7 use proteins from their host as processivity factor for their genome replication [17], [19].

The White Spot Syndrome Virus (WSSV) is a DNA virus that affects the shrimp aquaculture industry around the world [20], [21], [22]. It has been reported that this WSSV encodes its own DNA polymerase [23], [24], and we have demonstrated that WSSV ORF514 encodes a *bona fide* DNA polymerase. *In vitro*, this polymerase had a low processivity, although the presence of a PIP-box in its sequence and the absence of putative processivity factors in the virus genome suggest that it utilizes a host processivity factor [24], [25], [26]. We have recently reported the cDNA sequence, recombinant overexpression, purification and crystallization of the shrimp *Litopenaeus vannamei* PCNA [25], [27]. Moreover, others and ourselves have reported its gene expression during viral infection [25], [28],[29],[30]. Herein we report the x-ray structure analysis of the first crustacean recombinant PCNA (*Lv*PCNA) and a model where PCNA interacts with viral DNA polymerase PIP-box as an approach toward structural understanding this feasible interaction.

## Materials and Methods

### *Lv*PCNA purification and protein crystallization

Overexpression of recombinant *Lv*PCNA was carried using *E. coli* BL21 SI system and co-expression with chaperones was needed to obtain high yield of soluble recombinant protein. Metal affinity chromatography method was used for purification. Detailed description of overexpression, purification and *Lv*PCNA crystallization methods were previously reported [27].

Successful crystallization condition was: 300 mM CaCl_2_.2H_2_O, 100 mM sodium HEPES pH 7.5 and 30% *v/v* PEG 400. Thin hexagonal shaped crystals of approximately 0.1×0.6 mm were suitable for X-ray diffraction. The *Lv*PCNA crystal belonged to the C2 space group with unit-cell parameters *a* = 144.6 Å, *b* = 83.4 Å, *c* = 74.3 Å, β = 117.6° [27].

### X-ray data collection and crystallographic analysis

Data collection from *Lv*PCNA crystals was carried on beam line X4C of the National Synchrotron Light Source (NSLS), Brookhaven National Laboratory (BNL, Upton NY, USA), using a MarCCD 165 detector. The complete data covered 140° in 280 images, it was split and integrated independently using XDS and scaled together by XSCALE [31]. The phases were obtained by molecular replacement in PHASER [32] using an homology model of the *Lv*PCNA amino acid sequence (GenBank JN546075.1) as previously reported [25], based on the three-dimensional structure of human PCNA (PDB entry 1VYM) [33]. *Lv*PCNA refinement was carried out using the programs PHENIX [34]. Since the resolution was 3 Å, rigid body refinement and non-crystallographic symmetry between the monomers were imposed during refinement and manual rebuilding was done in COOT using 2F_o_-F_c_ maps at 2 σ to adjust positions and rotamers [35]. The final structure was deposited in the Protein Data Bank with accession number 4CS5.

### Molecular docking of WSSV DNA polymerase PIP-box into *Lv*PCNA crystal structure

In order to visualize if *Lv*PCNA could recognize WSSV DNA polymerase via its putative PIP-box, we performed a docking analysis using the software MOE 2102.10. The amino acid corresponding to the PIP-box from WSSV DNA polymerase was modeled by homology from residue 382 to 401, with the amino acid sequence ERAIGQHKILYYDIETTDKD. This template was selected by similarity with the sequence of a PIP-box peptide from Flap endonuclease 1 in complex with PCNA (PDB 1UL1)[36]. The final model for WSSV DNA polymerase PIP-box was refined from 25 intermediate models under the default parameters of the MOE homology modeling protocol using the CHARMM27 force field for energy minimization. The PIP-box binding site was defined from the resolved coordinates of *Lv*PCNA based on sequence identity on a multiple sequence and structural alignment of several PCNA crystallographic structures in complex with a PIP-box peptide or protein. A stochastic search of the best-fitted positions of the WSSV PIP-box peptide over the *Lv*PCNA pocket was done using the MOE Dock platform under the Induced Fit protocol. Ligand placement was performed using the Alpha Triangle method and the London dG scoring function for at least 80,000 poses. From this output, 30 non-duplicate poses were retained for further refinement used to relax the poses by 500 iterations with the Force field scheme and the Affinity dG rescoring function under the CHARMM27 force field. Duplicates from the refinement process were removed and the best scoring 30 poses were retained for further analysis. The final file was used for elaboration of figures and diagrams using CCP4mg [37], [38].

## Results and Discussion

### Determination of the *Lv*PCNA structure

Electron density maps calculated from the molecular replacement initial model showed good coverage of the backbone and followed the alpha helical trace of the protein. *Lv*PCNA had the cognate fold comprised by β-α-β_5_-α-β-β-β-IDCL-β-α-β_5_-α-β-β-β topology with pseudo symmetry within each monomer. After several cycles of refinement in PHENIX and manual rebuilding in COOT, both *R-work* and *R-free* dropped, suggesting that the refinement strategy was correct. Final refinement values were **R_work_** 0.2648 and **R_free(5%)_** 0.3108 (Table 1).

**Table 1:** Data reduction and refinement statistics from *Lv*PCNA structure.

DATA SET			*Lv*PCNA	
**Space group**			*C*2	
**Unit-cell parameters (Å)**			*a* = 144.57, *b* = 83.38, *c* = 74.31 β = 117.6°_	
**Data collection**				
	Resolution range (Å)		40.0–3.0 (3.1–3.0)	
	Unique reflections		14802 (1128)	
	R*_meas_* §		0.094 (0.415)	
	Completeness (%)		93.3 (97.0)	
	*I*/σ(*I*)		10.69 (3.57)	
	Redundancy		2.96 (3.10)	
**Refinement statistics**				
	R_work_/R_free(5%)_		0.2648/0.3108	
	R.M.S.D. form ideal			
		Bond length (Å)	0.01	
		Bond angles (°)	1.489	
	Ramachandran plot, residues in			
		Most favored regions	666(87.32%)	
		Additionally allowed regions	83(10.83%)	
		Outliers	14(1.85%)	

Values in parenthesis represent the statistics at the highest resolution bin.

§ R*_meas_* is a redundancy-independent version of R*_symm_*, R*_meas_* = ∑*_h_* √n*_h_*/n*_h_*−1 ∑^nh^_i_|Î_h_−I_h,i_|/∑*_h_* ∑^nh^_i_I_h,i_, where Î_h_ = 1/n_h_ ∑^nh^_i_I_h,i_.

**Figure 1:**
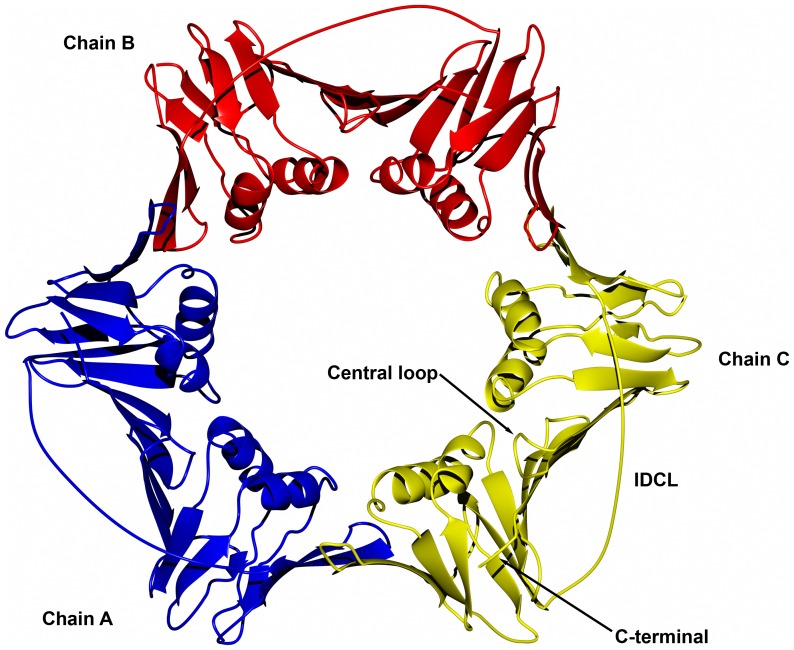
Crystal structure of *Litopenaues vannamei* PCNA. The PCNA molecule is arranged as homotrimer and each monomer is shown in different color. The most important parts for protein-protein interaction of each monomer: Interdomain Conecting Loop (IDCL), Central Loop and C-terminal are labeled.

**Figure 2:**
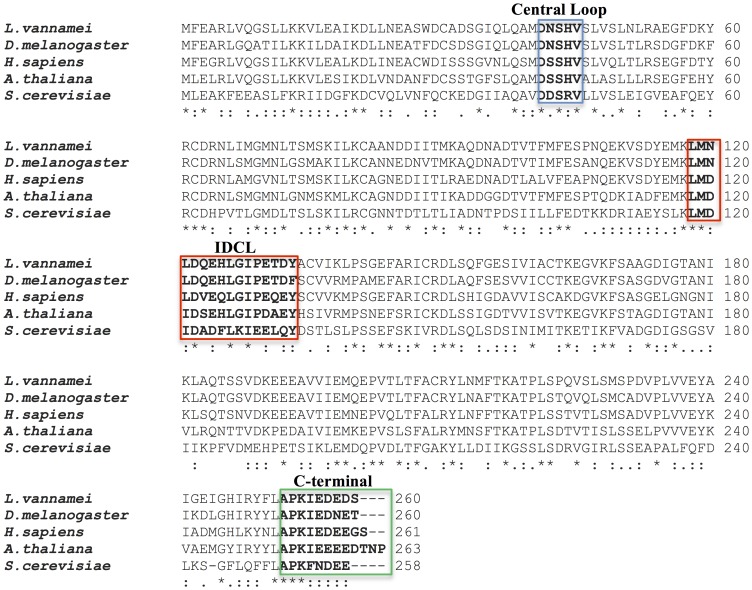
Amino acid sequence alignment of PCNAs. The figure shows the high identity and similitude of *L. vannamei* PCNA with other species. Important domains for PCNA-protein interactions: Central Loop, Inter-domain Connector Loop (IDCL) and C-terminal are in colored boxes (blue, red and green respectively) and tagged.

**Figure 3:**
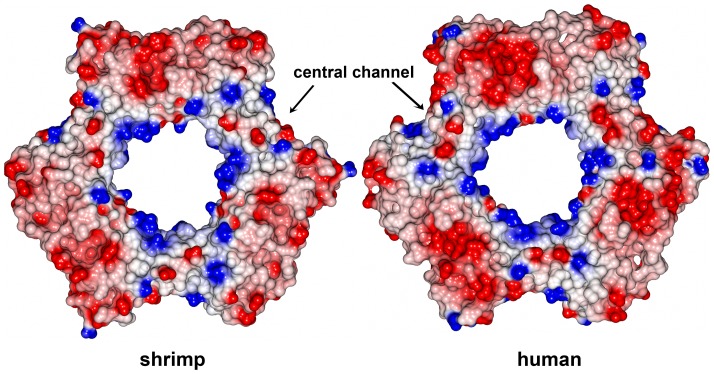
Electrostatic surface view of PCNA from shrimp *Litopenaeus vannamei* and human. The positively charged regions are colored in blue and negatively charged regions in red. The central channel is identified as a highly positive charged hole where the double-strand DNA can slide through it.

To determine the quaternary structure of LvPCNA we run this purified protein at 1mg/ml into a Superdex 200 size-exclusion chromatography column and compared its elution profile with known molecular-mass standards. LvPCNA eluted in a complex of approximately 90 kDa, indicating that this protein assembles as a trimer in solution [27]. Accordingly to this previous result the molecular replacement found a trimer in the asymmetric unit. The backbone cartoon shows the canonical structure and although the IDCL (residues 117–133) had poor electron density, the density was conclusive to include the coordinates of those residues in the final model (Figure 1). *Lv*PCNA amino acid sequence is highly conserved among species (Figure 2) and is structurally similar when compared with *Drosophila* PCNA [39], as it had a root mean square deviation (RSMD) of 0.5 Å for the α-carbon backbone. The central hole is highly positive charged as shown in Figure 3 and has a diameter of 30.5 Å, large enough to accommodate the double helical DNA and slide freely on it.

### Construction of *Lv*PCNA-WSSV PIP-box model

**Figure 4:**
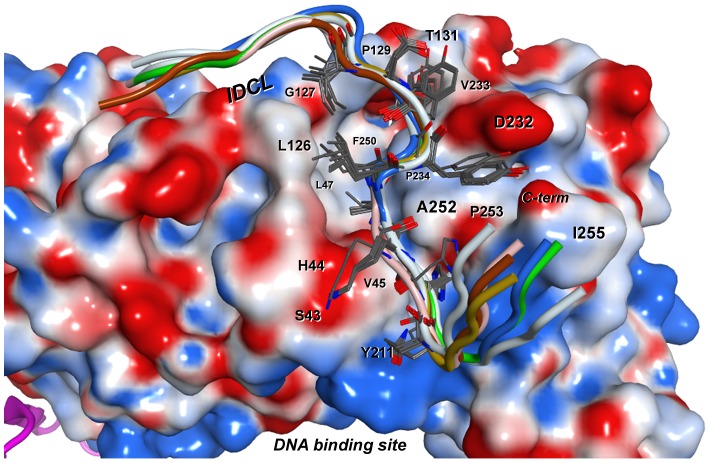
Aligment of best-scoring models of PCNA-WSSV PIP box complex from docking. The model shows the final seven poses for the PIP-box peptide (cartoon) docked into the binding site of *Lv*PCNA (surface representation). Tagged residues are from PCNA and form the cavity for peptide interaction. Side chains of the consensus PIP-box residues are shown as gray lines.

A peptide sequence containing the WSSV DNA polymerase PIP box was modeled and docked into the crystallographic structure of shrimp PCNA, which is its natural host. The docking of PIP-box peptide into the *Lv*PCNA binding site was carried out at the cognate region but without constraints to a specific position within the pocket in a stochastic approach. It is remarkable that the docking algorithm led to seven similar poses for the PIP-box peptide into the pocket between the 30 best-scoring ones (Figure 4). All this poses have an average RMSD of 2.2 Å for the α-carbon atoms of the entire peptides.

The peptide corresponds to a region of 20 amino acids from residues 675 to 694 of the WSSV DNA polymerase ORF (GenBank NP_478036). The peptide adopts an extended structure with a single helical turn at the center of the consensus sequence QHKILYY, very similar to other PIP-box peptides. This cognate structure is seen in most PIP-box peptides, even in those which showed a distinct pattern of contacts with a PCNA, such as in the translesion polymerases (Polη, Polι, and Polκ) and PCNA in humans [40]. It seems that these differences in amino acid sequence and contacts is the major way to determine the affinity of a PCNA partner, and so the decisive process over the DNA molecule [41].

**Figure 5:**
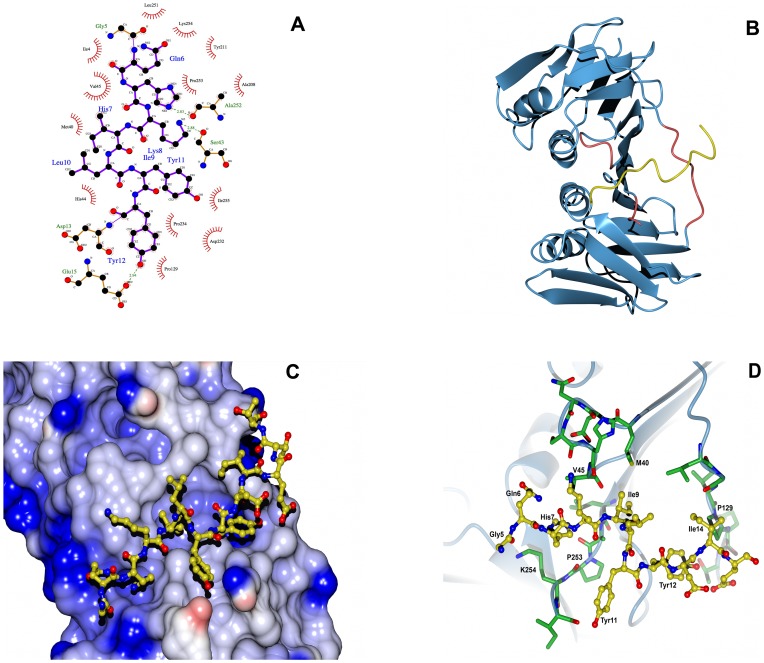
Model of *Lv*PCNA bound to WSSV PIPbox-peptide. In all figures the peptide was shorten to the consensus sequence GQHKILYYDIE that makes contact with *Lv*PCNA. Panel A shows a LigPlot where the peptide interacts with *Lv*PCNA through polar contacts (green dotted lines) and hydrophobic interaction (). Panel B shows a cartoon of the peptide (yellow) posed on a *Lv*PCNA monomer (blue), in red are identified the three region that participate in protein-protein interaction. In panel C, a surface image of *Lv*PCNA shows the hydrophobic pocket where the WSSV PIPbox-peptide (yellow) is attached. In panel D, residues that participate in *Lv*PCNA-peptide complex are tagged, side chains of residues from IDCL, Central Loop and C- terminal are green colored and the peptide residues are yellow colored.

The interactions between the PIP-box peptide and *Lv*PCNA are shown schematically as a LigPlot diagram (Figure 5, panel A) [42]. The PIP-box peptide interacts within each PCNA monomer almost in the internal symmetry axis and almost perpendicular to the IDCL loop as shown in cartoon (Figure 5, panel B). However, this is a tight packing cavity as obtained by docking, where mostly hydrophobic interactions are leading the binding, the hydrophobic cavity is represented in a surface image where the PIP-box peptide is positioned and drawn as sticks (Figure 5, panel C). This pocket comes mainly from the IDCL (G127, P129, T131), central loop (S43, H44, V45, L47) and from C-terminus (F250, L251, A252, P253, I255) residues.

The interaction between peptide and PCNA is mainly hydrophobic, only the H7 and K8 residues from the peptide make polar contacts with PCNA residue A252 and S43, respectively (Figure 5, panel A) and some intra-molecular interactions were found within the PIP-box peptide. Main hydrophobic contacts are between PIP-box residues G5, Q6, Y11 and *Lv*PCNA C-terminal domain L251, K254, P253, I255. The *Lv*PCNA central loop residues M40, V45, H44 make hydrophobic contacts with I9, L10 of the PIP-box and only P129 *Lv*PCNA IDCL residue makes hydrophobic contact with PIP-box Y12, the side chains of these residues are shown in figure 5, panel D.

**Figure 6:**
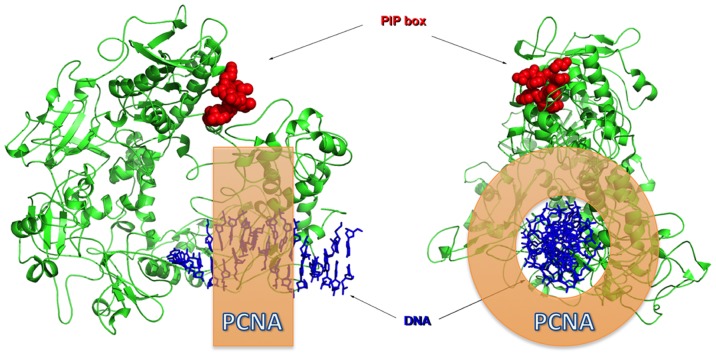
Proposed interaction between LvPCNA and WSSV DNA polymerase. Lateral and frontal view of the theoretical model of the polymerase is depicted with DNA. The PIP box is shown in red. PCNA is shown as an orange ring.

One feature observed during the docking process is that the algorithm produces several solutions or poses of the peptide into *Lv*PCNA, and the internal peptide sequence Q6-HKILYYD-I14 has an RMSD smaller than 1 Å for those poses (Figure 4). This ensures that the computational docking is consistent and reliable, until further confirmation by X-ray crystallography studies of the complex *Lv*PCNA with PIP-box peptide. To further envision the interaction between *Lv*PCNA and WSSV DNA pol, a theoretical model of the polymerase was built around DNA (Figure 6) and a ring with the average radius of the PCNA was drawn for an estimation of the interaction and closeness of both proteins. In this model the PIP box of WSSV DNA pol is in a position that indicates that upon a conformation change it could interact with *Lv*PCNA. Whether a conformational change occurs in WSSV DNA pol is necessary to produce the a tight interaction is something to be further explored.

## Conclusions

The crystal structure of the *Lv*PCNA has the expected trimeric ring shape, consistent with most of the eukaryotic PCNA reported. The results from docking suggest that WSSV polymerase has the capacity of binding the *Lv*PCNA in the same way that most PCNA binding proteins do. This possible interaction is predicted as hydrophobic which has to be considered when proved experimentally to elect the correct method. Despite the experimental phase of this interaction remains to be carried, it could lead to a future investigations toward generate an antiviral strategy that could prevent or disrupt this protein host-pathogen interaction, resulting in poor viral DNA replication and diminishing the pathogenicity of WSSV.
